# “Unsteady Gait”: An Unusual Presentation of Extrapulmonary Tuberculosis

**DOI:** 10.7759/cureus.9078

**Published:** 2020-07-08

**Authors:** Andrew Talon, Ericka Charley, Arnold N Forlemu, Dale Stern

**Affiliations:** 1 Internal Medicine, Creighton University Arizona Health Education Alliance/Valleywise Health Medical Center, Phoenix, USA; 2 Internal Medicine, Creighton University School of Medicine/St. Joseph's Hospital and Medical Center, Phoenix, USA

**Keywords:** central nervous system tuberculosis, myelopathy, spinal tuberculosis, tuberculosis, intramedullary tumor, spinal cord tumor surgery, intramedullary spinal cord tuberculoma, multidrug-resistant antituberculosis

## Abstract

Intramedullary tuberculoma (IMT) is a rare form of spinal cord tuberculosis (TB). Unlike Pott’s spine, IMT is without osseous involvement and is indolent. These features may account for why the diagnosis is often overlooked as a cause of compressive myelopathy. Our case is unique in that we discuss an unusual presentation of a patient who presented with gait disturbance as the first symptom of spinal cord TB without foci of TB infection elsewhere. The patient’s neurological symptoms improved with surgery and multidrug-resistant antituberculosis treatment. Although MRI is the preferred modality to characterize IMT, findings may be nonspecific, ultimately requiring biopsy. When IMT is diagnosed and managed appropriately, it carries a good prognosis. An interdisciplinary approach would provide the best outcomes.

## Introduction

Spinal cord involvement is estimated to account for less than 1% of all tuberculosis (TB) infections [1]. An intramedullary tuberculoma (IMT) is a rare form of spinal cord TB. Unlike Pott’s spine, a form of vertebral osteomyelitis that presents with back pain, IMT is often indolent and will only cause neurologic symptoms when cord compression is apparent [1]. These features may account for why the disease is often overlooked as a cause of myelopathy with regard to spinal cord masses. As such, the diagnosis and treatment of IMT is frequently delayed due to the rarity and atypical symptoms. Without timely treatment, neurologic sequelae can become debilitating and potentially irreversible. Here, we report an unusual case of cervical IMT as the primary manifestation of TB.

## Case presentation

A 40-year-old Filipino female presented with complaints of difficulty walking. Two months prior to presentation, she developed descending paresthesia from the chest down to both her feet with progressive asymmetrical weakness in her right hand and both lower extremities. The symptoms progressively worsened, and she began to use a front-wheel walker. She reported no constitutional or pulmonary symptoms. She had been working at a group home for the past five years. She denied history of exposure or prior TB infection. She did receive the bacille Calmette-Guérin (BCG) vaccine as a child. 

On examination, her upper and lower limb muscle strength was 5/5 bilaterally with normal tone and deep tendon reflexes. She had no sensory impairment. Gait was cautious. Inspection of her back revealed no erythema or other skin changes. There were no areas of focal tenderness on spine exam. The rest of her exam was unremarkable. Her complete blood count, electrolytes, B12 level, thyroid-stimulating hormone level, and renal and liver function tests were normal. Human immunodeficiency virus testing was negative. Her chest x-ray was normal. 

**Figure 1:**
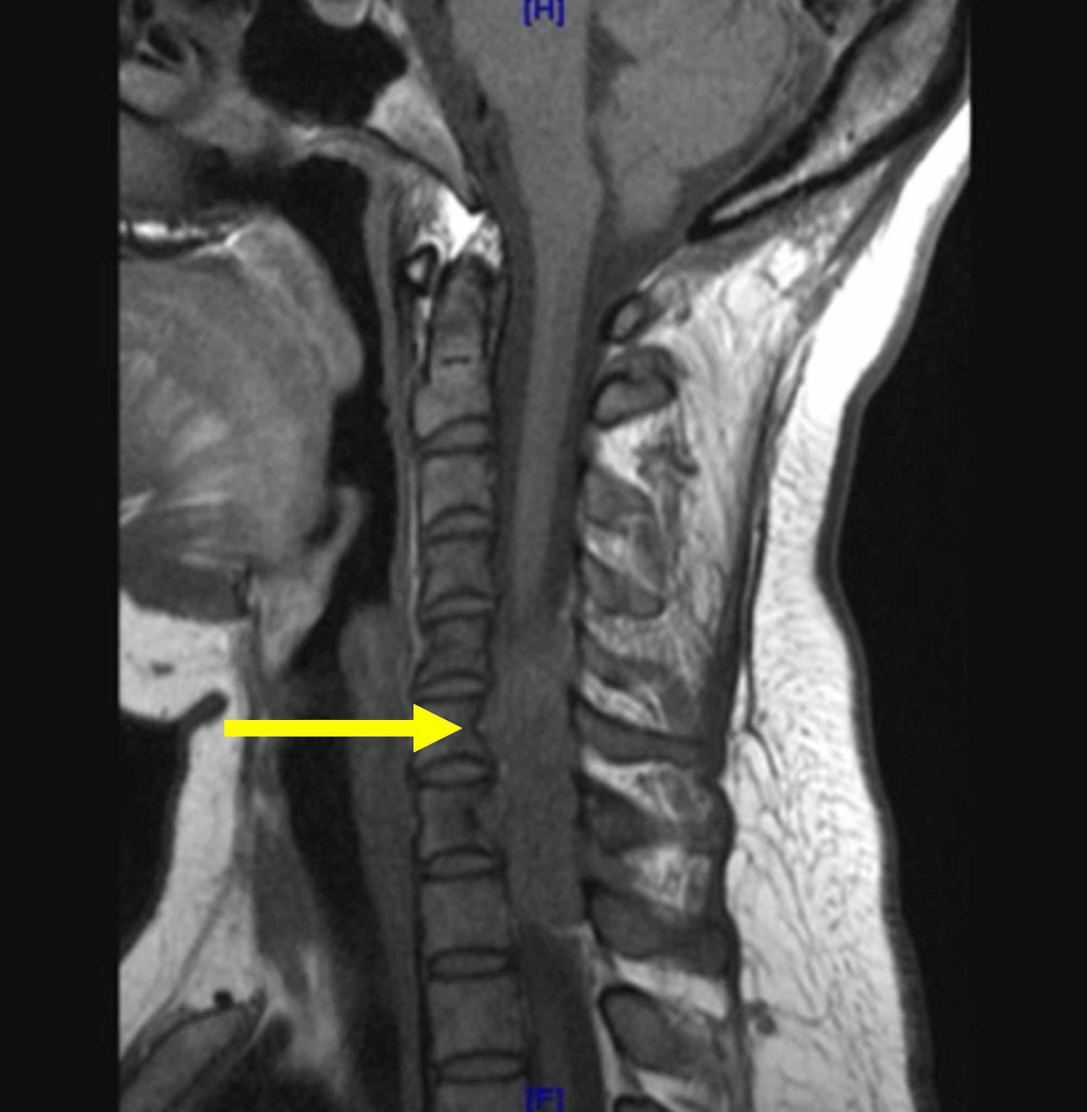
MRI cervical spine. Left epidural enhancing mass measuring 1.4 x 3.5 x 4.6 cm causing severe spinal cord compression from C6-T1, with infiltrative spread into the paravertebral soft tissues.

An MRI of the cervical spine revealed an epidural mass at C6-T1 on the left side of the spinal canal with severe cord compression (Figure 1). Due to concern for a possible malignant peripheral nerve sheath tumor, a core biopsy of the cervical spine was pursued. Biopsy revealed no evidence of malignant cells but instead showed granulomatous inflammation with rare acid fast organisms identified on acid-fast bacilli (AFB) stain. Quantiferon TB was positive. Mycobacterium tuberculosis polymerase chain reaction (PCR) and sputum AFB were negative. AFB cultures showed no growth.

**Figure 2:**
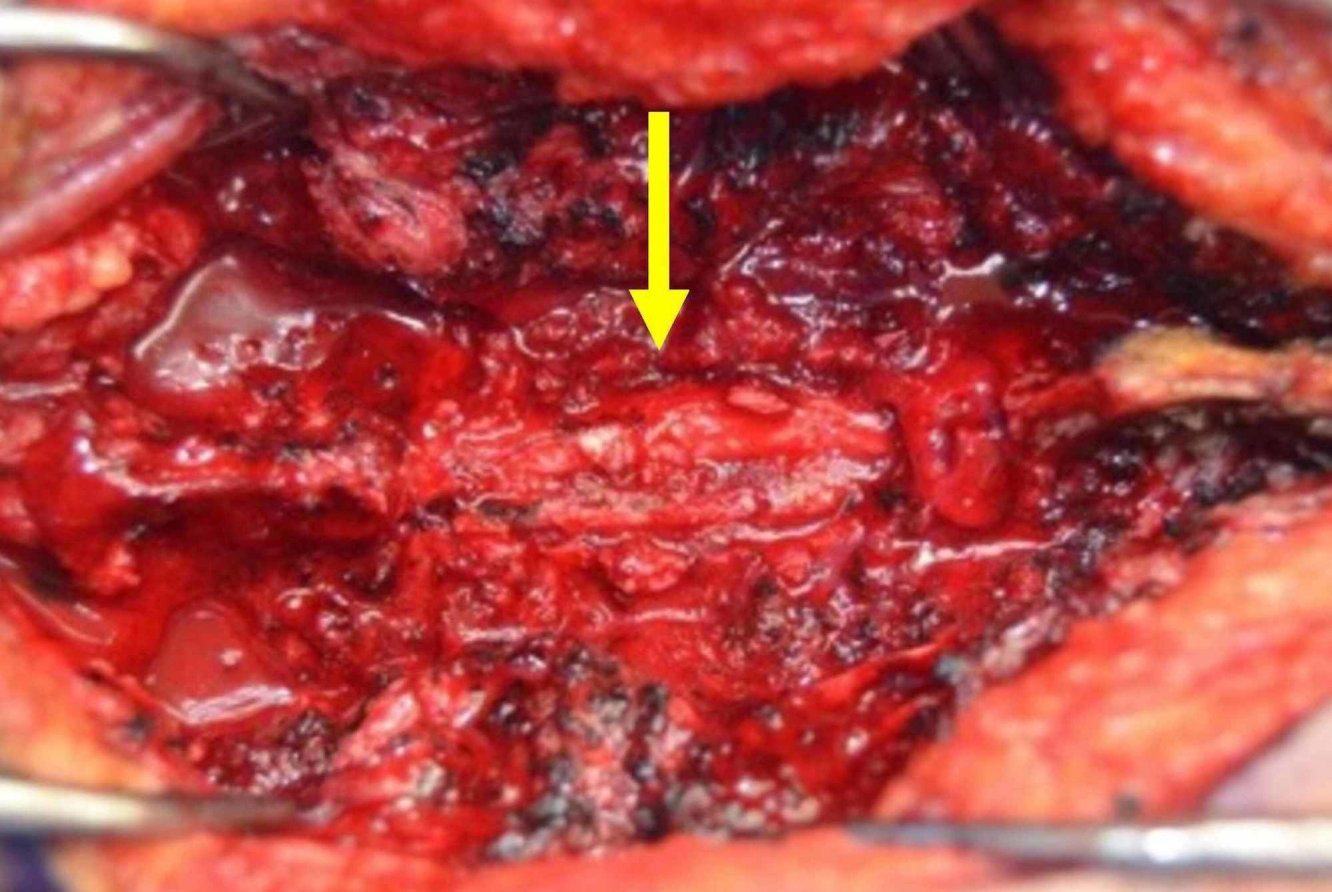
Tuberculoma found outside the dura between spinal levels C5-T1.

**Figure 3:**
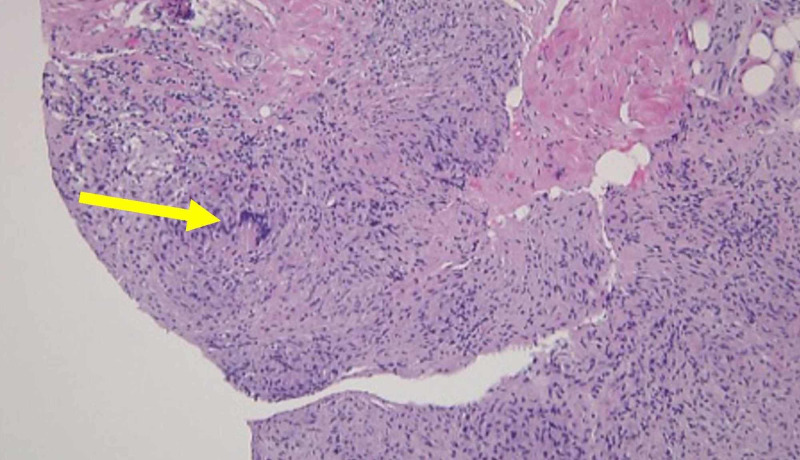
Histopathology of the intramedullary mass shows granulomatous inflammation, fibrosis with small foci of necrosis, and acid-fast bacilli.

Neurosurgery was consulted, and surgery was planned accordingly. The patient underwent decompressive laminectomy with segmental fixation, revealing a fleshy gray rubbery mass found outside the dura between spinal levels C5-T1 (Figure 2). Histopathology of the mass showed granulomatous inflammation, fibrosis with small foci of necrosis, and AFB (Figure 3). The patient was empirically treated for multidrug-resistant (MDR) TB and started on isoniazid (INH) 300 mg/day, rifampin 600 mg/day, pyrazinamide 1,500 mg/day, ethambutol 1,200 mg/day, moxifloxacin 400 mg/day, and amikacin 875 mg/day. Pyridoxine at 50 mg/day was also given. Following six months later, the patient could walk without a walker with a stable upright gait. 

## Discussion

IMT is estimated to account for only 2% of all central nervous system (CNS) TB infections [1]. When it does present, it is typically in the thoracic level of the spinal cord due to contiguous spread from the lungs [2]. The majority of cases are usually associated with foci of TB infection elsewhere [3]. TB can present as an extradural soft tissue mass compressing the spinal cord and mimic other intramedullary lesions. The differential diagnosis should include common spinal cord tumors such as ependymoma, astrocytoma, and lymphoma. Our case is unique since the patient had no systemic signs or radiographic evidence of TB and presented with a cervical rather than thoracic spine tuberculoma. The clinical presentation of an IMT varies by the level of spinal cord involvement, with the most common symptoms related to spinal cord compression, including lower limb weakness, paresthesia, and urinary and bowel incontinence [4]. MRI is the preferred modality to characterize IMTs for further treatment and planning [3]. A defining characteristic includes a “target sign”, indicative of development of caseating necrosis, which is described as central hypointensity surrounded by rim enhancement [4]. This key finding aids in differentiating IMT from other intramedullary masses. However in our case, MRI findings were nonspecific. Confirmation of the disease requires biopsy demonstrating AFB on microscopy or isolate culture [1]. PCR has currently been preferred as it allows for a more rapid diagnosis and greater sensitivity [1]. The mainstay of medical treatment should include isoniazid, rifampin, pyrazinamide, and either ethambutol or streptomycin [5]. Currently, there is no known optimal duration of antituberculosis treatment for IMT. Combination therapy of RIPE (rifampin, isoniazid, ethambutol, and pyrazinamide) for two months followed by combination of isoniazid and rifampin for a total period of 6, 9, 12, or 18 months is the most frequent protocol used for treatment of spinal TB [1]. A large multicenter retrospective study in Korea showed that radical surgery in conjunction with antituberculosis therapy was a significant prognostic factor for recovery [6].

Due to our patient previously residing in a highly endemic region for MDR TB and her community exposure risk, she was treated empirically as supported by *WHO treatment guidelines for drug-resistant tuberculosis* [5]. The role of ethambutol in RIPE therapy is to prevent drug resistance; however, the medication penetrates poorly into the CNS. Adding an alternative drug, such as a fluoroquinolone, is recommended for CNS penetration until drug susceptibility results are known. Amikacin was added as an additional bactericidal agent in case the organism is resistant to isoniazid, rifampin, or pyrazinamide [5]. 

Susceptibility testing options include culture, nucleic acid amplification tests, microscopic observation drug-susceptibility assay, and DNA sequencing. Testing through culture is standard of care but may take 8-16 weeks [3]. In our case, we ordered resistance testing and started treatment pending the results. 

## Conclusions

The authors present a case report of an IMT. Although IMT is a rare diagnosis for spinal cord masses, considering the diagnosis of TB and carefully assessing risk factors can avoid delays in its diagnosis and management. IMT carries a good prognosis when treated effectively with a combination of antituberculosis treatment and surgical resection, yet consequences of a missed or delayed diagnosis may prove debilitating. 
